# A framework for scientific advice on health: EuSANH’s principles and guidelines

**DOI:** 10.1186/1478-4505-11-6

**Published:** 2013-02-22

**Authors:** Antonio Sarría-Santamera, Eert J Schoten, Theodora M M Coenen, Louise J Gunning-Schepers, André Pauwels, Susanne V Allander, Miroslaw J Wysocki, Marius Ciutan, Carlos Segovia

**Affiliations:** 1 Instituto de Salud Carlos III, Agency for Health Technology Assessment ISCIII, Calle Sinesio, Delgado 4-6, 28029, Madrid, Spain; 2 Health Council of the Netherlands, PO Box 16052, 2500 BB, The Hague, The Netherlands; 3 Superior Health Council of Belgium, Rue de L’Autonomie 4, 1070, Brussels, Belgium; 4 Läkemedelsverket, Medical Products Agency, P.O. Box 26, SE- 751 03, Uppsala, Sweden; 5 National Institute of Public Health, Str. Chocimska 24, 00-791, Warsaw, Poland; 6 National School of Public Health Management and Professional Development, Vaselor 31, 021253, Bucharest, Romania; 7 National Instituto de Salud Carlos III, Calle Sinesio Delgado 4-6, 28029, Madrid, Spain

**Keywords:** European science advisory network for health, Guidelines, Methodology, Policy, Scientific advice

## Abstract

**Background:**

Society expects politicians to make sound decisions by bringing the best evidence to bear on the health problems in question. Performing this task requires access to independent sources of sound scientific advice. The European Science Advisory Network for Health (EuSANH) is a network of national science advisory bodies in Europe which are active in the field of health and provide independent scientific advice to their authorities. The EuSANH addressed this question in a European project.

**Methods:**

Guidelines and principles for producing sound advice have been formulated after international comparative evaluations and extensive discussions among participants of the EuSANH-ISA project with input from international experts.

**Results:**

A framework for scientific advice on health has been produced.

**Conclusions:**

This framework will ensure a uniform approach and thus opens possibilities for collaboration between science advisory bodies.

## Background

### Science and policy

In society there is a clear and growing recognition of the role of scientific and technical knowledge in advancing human health. However, individual scientists usually do not speak with one voice, the outcomes of their research often involve uncertainties, or they may address issues which have no direct societal applications or implications. Practical issues of relevance to society are the very points of departure and reference for policy makers. These two approaches do not necessarily link-up. Citizens expect politicians to make sound decisions by bringing the best evidence to bear on the problems in question. Carrying out this task requires access to independent sources of sound scientific advice. But what is ‘sound’? The European Science Advisory Network for Health (EuSANH; participating countries can be found at http://www.eusanh.eu) addressed this question in the three-year project entitled Improving Science Advice for Health in Europe (EuSANH-ISA). During this project a common methodological framework for scientific advice, a first joint scientific advice and a sustainable EuSANH structure have been developed. This paper is an executive summary of the full methodological framework with principles and guidelines for scientific advice and is meant to draw attention to these guidelines.

### Bridging the gap

The worlds of science and policy have their own position, language, and dynamics. How can the two meet? When one thinks in terms of distinct and contrasting concepts characterizing each world, such as facts versus values, objectivity versus subjectivity, or truth versus power, scientific advice seems almost a paradox [1]. Studies of scientific advising have shown, however, that it is not possible to draw sharp boundaries between facts and values [1]. For complex policy questions input from different scientific fields is required. Science advisory bodies can combine and translate them into policy recommendations. Discussion among scientists, as well as between scientists and policy makers, is one of the keys to the success of the advisory process. Scientific knowledge and information must always be debated and valued within the context of political problems. This usually involves a process of gradually adjusting divergent or complementary scientific viewpoints. At the same time, however, standards of adequacy for scientific evidence and inference are applied. As long as this is done in a transparent manner, the conclusions and recommendations of Advisory Committees will be viewed as highly credible. Thus, a firm scientific underpinning may be provided for public policy development.

Important though scientific advice may be, measures to be taken always also have political, economic, social or cultural aspects that must be considered. That is where scientific advice ends and policy begins. Weighing these aspects is up to policy makers and politicians, and takes place within the context of political and societal values, beliefs, and objectives.

### Sound scientific advice

Building on the general definition of scientific advice [2], scientific advice on health is defined as the solicited or unsolicited analysis of a defined public health, health care or health policy problem, based on updated scientific knowledge, considering also relevant expert judgment, practical experience, and ethical, cultural and societal values and implications, with conclusions and recommendations for health policy.

The principles and guidelines contained in this framework address how high-quality science advisory reports should be produced which may be effectively used in policy decision making. They have been formulated after international comparative evaluations and extensive discussions among participants of the EuSANH-ISA project with input from international experts [3]. Also, related frameworks developed by other organizations have been considered in these discussions [2,4-12]. The principles and guidelines are not only relevant as a quality seal to current EuSANH members, however. Because all national and international health authorities face similar problems and are expected to base their decisions and programmes on the best available evidence, this methodological framework may help any advisory body in providing sound scientific advice. A summary of the principles and guidelines is given in Table 1. More detailed information can be found in the full publication, A Framework for Science Advice on Health: Principles and Guidelines [13]. Examples of the framework are the reports on ‘determinants of a successful implementation of population-based cancer screening programmes’ and ‘childhood leukaemia and environmental factors’ [14-16]. The impact of both reports will become clear in the coming period.

**Table 1:** Framework for scientific advice on health

**Steps**	**Principles**	**Guidelines**	
**Framing the issue**	**Need**	1	Policy makers and science advisors should regularly discuss emerging issues requiring advice
		2	Science advisors should do so in interaction with the health research community
		3	In formulating a request for advice, policy makers and science advisors should determine in close cooperation the set of questions to be addressed
		4	Science advisors should discuss with policy makers whether a European or international perspective is appropriate
**Planning the process**	**Timeliness**	5	In framing the issue policy makers and science advisors should discuss the scope and duration of the task, considering the stage within the policy making process when scientific advice is needed
		6	The advisory body should develop operation procedures to manage the entire advisory process
**Drafting the report**	**Credibility**	7	Select committee members on the basis of professional excellence and with an appropriate range of expertise
		8	Select committee members who reflect the diversity of scientific opinions
	**Independence**	9	Screen for conflicts of interest in order to avoid advocacy
		10	Committee members should carry out their deliberations in closed meetings in order to avoid political and special interest influence
		11	The Committee should be responsible and accountable for the final report
	**Relevance**	12	Consider adding a policy maker to the Committee as an official observer
		13	Consider organizing stakeholder hearings
		14	Where appropriate, specify ethical or legal principles involved
	**Transparency**	15	Specify data and data sources used in producing the report
		16	Document and explain all assumptions made and methods used in interpreting and synthesizing the data
		17	Identify and describe all uncertainties involved
		18	Indicate where and how expert judgment is applied
**Formulating the recommendations**	**Feasibility**	19	Consider the potential consequences of the recommendations made to policy makers
		20	Where appropriate, identify policy options based on data and research evidence
**Reviewing the report**	**Quality**	21	The final draft report should undergo an independent peer review
		22	Guarantee continuity in producing advisory reports on similar issues
		23	Check whether the final draft report is consistent with other reports of the advisory body
		24	Specify the response to the comments made in the peer review
**Publishing the report**	**Openness**	25	Make the report publicly available
		26	Where more active dissemination is required, issue press statements, press releases or press briefings
		27	Where more clarification is required, organize meetings with policy makers and target groups
**Assessing the impact**	**Accountability**	28	There should be a follow-up procedure that monitors the policy makers’ actions in response to the advisory report
		29	The advisory body should regularly perform a (self)assessment, both of the impact of its reports and of its performance

More detailed information can be found in the full publication [13].

### Practical application of the framework

First, it must be emphasized that implementation of the guidelines presented here requires attention to specific circumstances, such as the legal or strategic position of the advisory body and the associated administrative traditions within which it has to operate. Put another way, each organization should examine how the guidelines can be operationalized in its own situation. Operationalizations may also vary depending on the issue under analysis. In fact, the phrasing of various guidelines, for example: “Consider …” or “Where appropriate, …” already explicitly invites advisory bodies to explore and compare alternative procedures. Second, the framework addresses the core business of advisory bodies, with regards to appointing multidisciplinary committees to advice on request of government agencies. Sometimes other working methods may be considered, such as a working conference or an advisory letter, where experts are consulted outside of a Committee setting. Many guidelines will then still provide valuable assistance. Finally, the framework should be considered from a dynamic perspective. In the coming years, all experiences and lessons learned should lead to regular updating and may lead to fine tuning or modifying the guidelines. In this context, the principles provide the robust architecture and will remain leading.

## Conclusions

This methodological framework will ensure a more uniform approach in Europe and thus opens possibilities for collaboration between national science advisory bodies. Since the scientific base of our policy decisions is the same, much of the process of science advice can be shared, even if the recommendations will always be particular to the country.

## Abbreviations

EuSANH: European Science Advisory Network for Health

## Competing interests

The authors declare that they have no competing interests.

## Authors’ contributions

AS and EJS designed the study, developed the outline, and contributed to the writing and revision of the principles and guidelines. TMMC: Helped to develop the outline, contributed to the writing and coordinated the work. LJG: Helped to develop the outline, and contributed to the writing and revision of the guidelines. AP, SVA, MJW, MC and CS: Prepared drafts versions of guidelines. All authors read and approved the manuscript.
